# Continuous coloured light altered human brain haemodynamics and oxygenation assessed by systemic physiology augmented functional near-infrared spectroscopy

**DOI:** 10.1038/s41598-017-09970-z

**Published:** 2017-08-30

**Authors:** A. J. Metz, S. D. Klein, F. Scholkmann, U. Wolf

**Affiliations:** 1 University of Bern, Institute of Complementary Medicine, Bern, Switzerland; 2 University Hospital Zurich, University of Zurich, Department of Neonatology, Biomedical Optics Research Laboratory, Zurich, Switzerland

## Abstract

Exposure to artificial coloured light is unavoidable in our modern life, but we are only just beginning to understand the impact of coloured light on human physiology. The aim of the present study was to determine effects of coloured light exposure on human systemic and brain physiology using systemic physiology augmented functional near-infrared spectroscopy (SPA-fNIRS). We measured changes in haemoglobin concentrations and tissue oxygen saturation in the left and right prefrontal cortices (L-PFC, R-PFC) by fNIRS, and also recorded skin conductance (SC), partial pressure of end-tidal CO_2_ (P_ET_CO_2_), and heart-rate variability variables. 17 healthy adults (median age: 29 years, range: 25–65 years, 6 women) were exposed to blue, red, green, or yellow light for 10 minutes. Pre-light and post-light conditions were in darkness. In the L-PFC the yellow evoked a brain activation. SC and P_ET_CO_2_ did not change during any of the coloured light exposures, but SC increased and P_ET_CO_2_ decreased for all colours (except green) in the post-light period. Changes in L-PFC haemoglobin concentration were also observed during the post-light period but have to be interpreted with care, because heart rate and SC increased while P_ET_CO_2_ decreased. The detected effects are potentially of high relevance for choosing room lighting and may possibly be applied therapeutically.

## Introduction

We are increasingly exposed to various sources of coloured light, such as advertisements and different kinds of video screens. All emit light of various spectral compositions impinging on our eyes and body. These coloured lights may induce specific physiological changes in our human body. The importance of the interaction between coloured light and human physiology is underlined by the existence of many therapeutic applications of light. For example, blue light treatment of neonatal jaundice^1^ and white and blue light treatments of seasonal affective disorder and depression^2–7^ are established medical applications.

Known systemic physiological effects include the suppression of *melatonin* secretion, a hormone initiating sleep, by blue and white light^8–14^, and an interaction between *cortisol*, a hormone associated with stress, and white light exposure^15^. Blue and white light have also been effective in altering the core body temperature^16^ and heart rate (HR)^16–19^, both of which fall in the late evening as part of the circadian rhythm. During the day, no effects of coloured light have been found on HR^20–25^ or body temperature^26^. Only a few studies assessed the effects of coloured light during the day. Some of these have reported about correlates of neuronal activity, which are sensitive to coloured light during the day: Blue light has altered the amplitude or latency of auditory evoked potentials in an auditory oddball tasks, measured by electroencephalography (EEG)^27–29^. EEG alpha and beta power have also been affected by coloured light^30–33^, but not always^29^. Several functional magnetic resonance imaging (fMRI) studies have reported increased blood oxygen level dependent (BOLD) responses when subjects performed a working memory task under blue light, compared to performing a task under another colour^34–36^. Blue light exposure has been found to increase cerebral tissue oxygen saturation in awake and resting subjects, assessed by functional near-infrared spectroscopy (fNIRS)^37^. Furthermore, effects were also observed during the night, e.g. the inhibition of EEG delta power, an indicator of sleep, in the late evening by blue light^17, 38, 39^.

Some studies on heart-rate variability (HRV) have shown that the high-frequency (HF) component of the HRV increased with higher fraction of blue light^22, 25, 40, 41^, while other studies did not^23, 42^. Red light was found to increase the low-frequency (LF) to HF ratio (LF/HF)^22, 43^, yet other results were ambiguous^25^ or contradictory^23, 42^.

The studies cited above point towards an effect of coloured light on human physiology, but many physiological variables remain unexplored, especially during the day. Studies on skin conductance^20, 22, 44–46^ have not found a clear and reproducible effect of coloured light on human physiology and no effects of coloured light on the arterial partial pressure of carbon dioxide (P_a_CO_2_) have been published. Together with the fact, that the fNIRS and HRV studies on this topic are limited in number and the results ambiguous, it motivated us to further investigate the effect of coloured light on human physiology.

Hence, the aim of this study was to assess possibly unknown effects of coloured light exposure on adult human subjects’ neuronal and systemic physiology. As outcome variables, we selected neuronal physiology by means of fNIRS, as well as systemic physiology, represented by skin conductance, end tidal CO_2_, P_ET_CO_2_, which correlates with P_a_CO_2_^47^, several variables of the HRV, and the HR. This is the first study employing systemic physiology augmented functional near-infrared spectroscopy (SPA-fNIRS) to assess the effect of coloured light exposure on human physiology. We hypothesized that the different colours blue, green, red, and yellow would affect the outcome variables differently.

## Results

### Skin conductance

**Figure 1:**
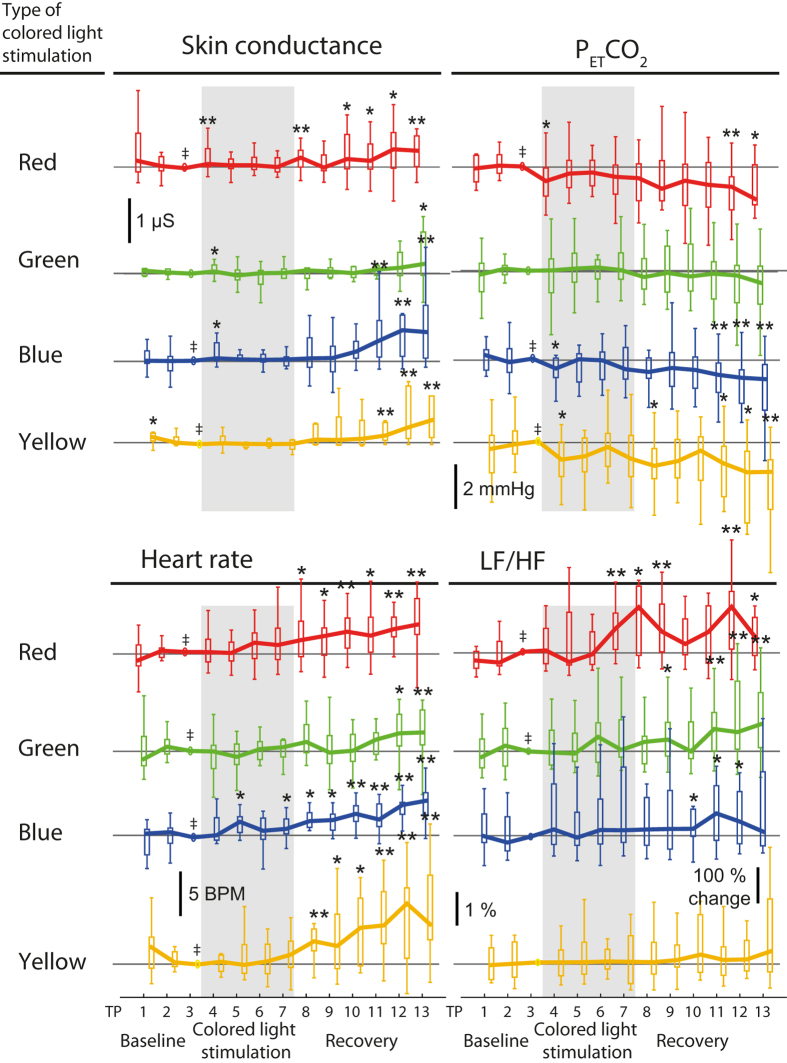
Paired changes of skin conductance (SC), P_ET_CO_2_, HR and the LF/HF ratio calculated from ECG data. Each panel includes a time series for each colour, as denoted. The time series are sub-divided into thirteen 2.5 min periods. The two bold vertical lines mark the light exposure period (Colour Stimulation). Time series are always normalized (subtracted, or for LF/HF divided by) to the last time point of the baseline (TP3). Hence the horizontal lines represent zero (one for LF/HF). ^‡^Indicate significance of a change throughout the whole times series (Friedman tests and FDR correction, SC: *p*_all_ < 0.03, P_ET_CO_2_: *p*_all_ < 0.05, HR and LF/HF: *p*_all_ < 0.01). Asterisks indicate a significant change of the marked time point with respect to TP3 (Wilcoxon signed rank tests, uncorrected). Outliers are not displayed. TP: time point, BPM: beats per minute. HR: heart rate. ECG: electrocardiogram.

Skin conductance increased for all colour conditions, although during green light exposure the increase was less pronounced and not significant after false discovery rate (FDR) correction^48^. This change was observed especially after light exposure in the recovery period (*p*_all_ < 0.01, green: *p*_all_ < 0.05, n.s. after FDR correction), no change was observed during the baseline and coloured light exposure itself (all *p*_col_ and *p*_recov_ > *α*_SC_). The changes did not depend on the colour condition (all *p*_betwc_ = n.s.). Refer to Fig. 1 and Table 3 for the detailed changes and significances. Please refer to the methods section for the explanation of the different *p*- and *α*-values.

### P_ET_CO_2_

For all colours, no changes in P_ET_CO_2_ were observed during baseline and coloured light exposure (*p*_col_ > *α*_CO2_). At the end of the recovery period P_ET_CO_2_ was decreased compared to during coloured light exposure for red, blue, and yellow (*p*_recov_ < 0.001), but not for green. The changes were, however, not dependent on the colour condition (all *p*_betwc_ = n.s.). Refer to Fig. 1 and Table 3 for the detailed changes and significances.

### Heart rate

Heart rate increased throughout all colour conditions (*p*_all_ < 0.001), and specifically during the recovery period (blue, yellow: *p*_recov_ < 0.001, red, green: *α*_HRV_ < *p*_recov_ < 0.05). No dependence on the colour condition was found (all *p*_betwc_ = n.s.).

### Heart-rate variability

From the HRV data, the total power, as well as the VLF power decreased throughout the complete times series (all *p*_all_ < 0.001), and specifically during the recovery period (*p*_recov_ < 0.001, except for VLF red, green and total power red: *α*_HRV_ < p_recov_ < 0.05, total power green: *p*_recov_ = n.s.). Normalized LF and HF powers (LF norm, HF norm) changed in the red and green light conditions. LF norm increased and HF norm decreased during red light exposure (*α*_HRV_ < *p*_col_ < 0.05), but not during the recovery period (*p*_recov_ = n.s.), whereas LF norm increased and HF norm decreased after green light exposure (*α*_HRV_ < *p*_recov_ < 0.05) and not during the light exposure (*p*_col_ = n.s.). Accordingly, the LF/HF ratio increased during red light and decreased after green light exposure. Decreases during light exposure were also observed for absolute HF values during red and blue light exposure (*α*_HRV_ < *p*_col_ < 0.05). No changes were observed for standard deviation of the consecutive heart-beat intervals (SDNN), but RMSSD (the root mean-square of sum of squares of differences between adjacent heart-beat intervals) decreased under the red, blue, and green light condition (*p*_col_ < 0.05), but only the blue condition was significant after FDR correction (*p*_col_ < *α*_HRV_). No dependence on the colour condition was found (all *p*_betwc_ = n.s.). Indication of the variable changes and Friedman test results are displayed in Table 2.

### fNIRS data

**Figure 2:**
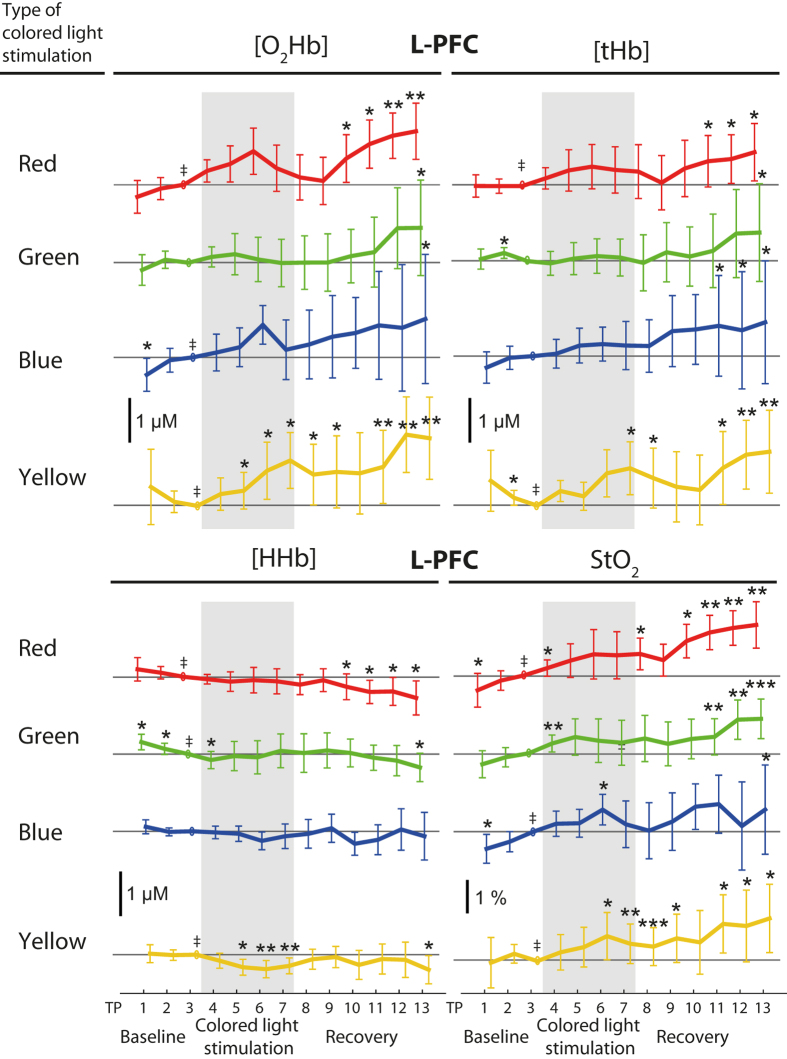
Paired fNIRS changes in the L-PFC evoked by light of different colours, i.e. red, green, blue, yellow. Each panel includes four time series, one for each colour condition, as denoted. The different time series are arbitrarily shifted for better visibility. All time series were normalized (subtraction) to the last time point of the baseline (TP3), thus this time point is always zero. Division of the time series, vertical bars, ^‡^ and asterisks are as in Fig. 1. Error bars represent the 95% confidence interval.

**Figure 3:**
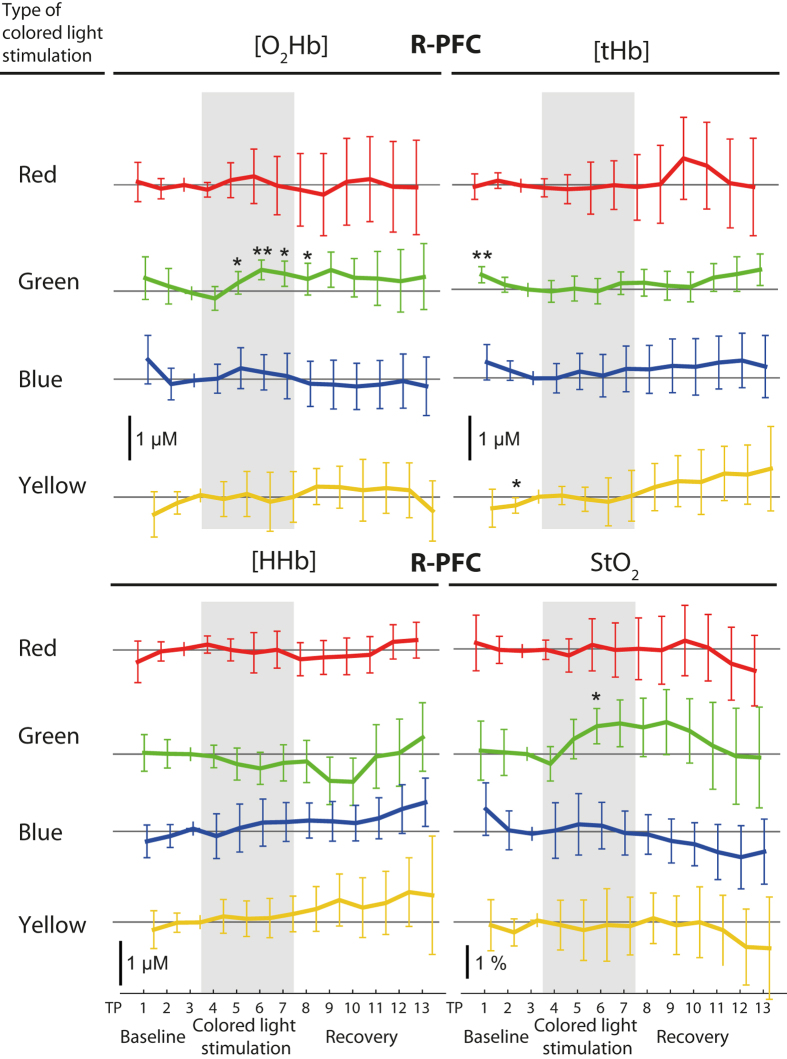
Paired fNIRS changes in the R-PFC evoked by light of different colours, i.e. red, green, blue, yellow. Each panel includes four time series, one for each colour condition, as denoted. The different time series are arbitrarily shifted for visibility. All time series were normalized (subtraction) to the last time point of the baseline (TP3), thus this time point is always zero. Division of the time series, vertical bars, ^‡^ and asterisks are as in Fig. 1. Error bars represent the 95% confidence interval.

Data recorded from the L-PFC changed over time in nearly all time series, contrary to the R-PFC, where no changes were observed. In the L-PFC, mostly, oxygenated and total haemoglobin concentrations ([O_2_Hb], [tHb]) and cerebral tissue oxygen saturation (StO_2_) increased while concentration of deoxygenated haemoglobin ([HHb]) decreased. When testing between baseline and coloured light exposure changes during yellow light exposure remained (yellow [O_2_Hb], [HHb], StO_2_: *p*_col_ < *α*_NIRS_, yellow [tHb]: 0.05 < *p*_col_ < 0.1). For all other colours, the changes occurred during the recovery period, or the specific Friedman tests for light exposure and recovery were not significant at all (blue [HHb], [tHb], StO_2_, *p*_col_ and *p*_recov_ = n.s.). See Fig. 2 and Table 3 for the changes and significances. The difference between coloured light exposure and baseline (TP7-TP3) were significant in-between colours for [O_2_Hb] (*p*_betwc_ < 0.05). Differences for the other variables and the differences between recovery and coloured light exposure (TP13-TP7) were not colour dependent (*p*_betwc_ = n.s.). The R-PFC showed a different response compared to the L-PFC: during the green coloured light stimulation a slight increase in [O_2_Hb] and StO_2_ could be observed (see Fig. 3), which, however, was not significant after FDR correction.

### Multidimensional mood-state questionnaire

For the multidimensional mood-state questionnaire (MDMQ), the Friedman tests did not reveal a main effect of colour on any of the scales. A trend was observed for wake-tired, (*p* < 0.1, *N* = 13), when excluding the data of the yellow condition. The Wilcoxon signed rank tests showed that subjects were more tired after red light exposure (*p* < 0.01, *N* = 14) and generally, including all colour conditions (*p* < 0.01, *N* = 57). No effects were observed for the other colour conditions (Table 1).

**Table 1:** Changes in the three scales of the multidimensional mood-state questionnaire for each colour condition and for all colours combined. Mean ± standard deviation; **p < 0.01 (Wilcoxon signed rank test).

Colour	Mood	Alertness	Calmness
Red	−1.07 ± 3.25	−1.93 ± 2.27/**	−0.38 ± 3.97
Green	−0.06 ± 2.57	−0.19 ± 2.37	0.06 ± 2.29
Blue	−1.53 ± 3.48	−1.47 ± 3.20	−0.60 ± 3.91
Yellow	−1.25 ± 3.62	−1.33 ± 3.99	0.08 ± 3.80
All	−0.95 ± 3.19	−1.19 ± 2.97/**	−0.21 ± 3.43

**Table 2:** Change of the HRV variables and the HR for each colour condition. Three changes are given, indicated by the time points (TP) in the second column. Note that the asterisks indicate significance according to the Friedman test but not the paired test for the displayed TP change. Results for the three Friedman tests for a change during colour stimulation (*p*_col_, in row TP7-TP3), during recovery (*p*_recov_, in row TP13-TP7), and for an overall change (*p*_all_, in row TP13-TP3) are indicated.

		Mean change ± SD of variable/Result of Friedman test			
		Red	Green	Blue	Yellow
Total power	TP7-TP3	−26.62 ± 73.70	−12.358 ± 68.72/*	−39.452 ± 58.38/*	−18.646 ± 53.95
[ms^2^ × 10^−3^]	TP13-TP7	−49.15 ± 103.06/*	−52.94 ± 96.05	−64.54 ± 56.10/***	−115.88 ± 102.13/***
	TP13-TP3	−75.77 ± 102.37/***	−65.30 ± 101.86/***	−103.99 ± 67.04/***	−134.53 ± 122.15/***
VLF	TP7-TP3	−26.759 ± 73.57	−12.342 ± 68.52/*	−39.112 ± 58.35/*	−18.016 ± 53.46
[ms^2^ × 10^−3^]	TP13-TP7	−48.63 ± 102.52/*	−52.78 ± 95.71/*	−64.12 ± 55.97/***	−116.19 ± 101.87/***
	TP13-TP3	−75.38 ± 101.76/***	−65.12 ± 101.44/***	−103.23 ± 66.92/***	−134.21 ± 121.69/***
LF	TP7-TP3	351 ± 641	87 ± 1329	−77 ± 986	−660 ± 1705
[ms^2^]	TP13-TP7	−362 ± 1160	24 ± 1369	−302 ± 1116	431 ± 1291
	TP13-TP3	−12 ± 1009	111 ± 1182	−379 ± 1320	−229 ± 2124
LF norm	TP7-TP3	7.41 ± 10.01/*	3.99 ± 12.97	8.15 ± 14.25	−1.49 ± 16.09
[%]	TP13-TP7	−0.82 ± 13.12	5.74 ± 14.33/*	0.48 ± 16.96	6.68 ± 9.79
	TP13-TP3	6.59 ± 13.03/***	9.73 ± 15.49/**	8.64 ± 19.37	5.19 ± 19.53
HF	TP7-TP3	−212 ± 363	−103 ± 437	−263 ± 301/*	30 ± 704
[ms^2^]	TP13-TP7	−164 ± 569/*	−190 ± 376	−117 ± 356	−117 ± 309
	TP13-TP3	−376 ± 715/**	−293 ± 575	−380 ± 597/**	−87 ± 832
HF norm	TP7-TP3	−7.41 ± 10.01/*	−3.99 ± 12.97	−8.15 ± 14.25	1.49 ± 16.09
[%]	TP13-TP7	0.82 ± 13.12	−5.74 ± 14.33/*	−0.48 ± 16.96	−6.68 ± 9.79
	TP13-TP3	−6.59 ± 13.03/***	−9.73 ± 15.49/**	−8.64 ± 19.37	−5.19 ± 19.53
LF_HF	TP7-TP3	25.69 ± 35.47/*	−0.06 ± 58.67	14.16 ± 61.91	−47.15 ± 119.75
[%]	TP13-TP7	24.97 ± 96.14	74.11 ± 137.90/*	37.69 ± 119.10	88.36 ± 155.50
	TP13-TP3	50.66 ± 90.15/***	74.05 ± 141.10/**	51.84 ± 123.95	41.20 ± 183.26
SDNN	TP7/TP3	4.72 ± 13.14	4.02 ± 15.86	−0.87 ± 15.81	−1.59 ± 17.65
[ms]	TP13/TP7	−2.54 ± 22.36	0.40 ± 17.59	2.66 ± 21.75	3.38 ± 17.76
	TP13/TP3	2.18 ± 18.09	4.42 ± 17.32	1.79 ± 17.95	1.79 ± 16.58
RMSSD	TP7-TP3	−3.95 ± 6.07	−1.68 ± 6.87	−3.94 ± 4.15/**	−2.49 ± 7.14
[ms]	TP13-TP7	−5.96 ± 9.98	−4.16 ± 9.85	−2.93 ± 9.80	−4.24 ± 10.12
	TP13-TP3	−9.91 ± 11.75/*	−5.84 ± 10.86/*	−6.87 ± 8.65/**	−6.73 ± 11.49
HR	TP7-TP3	1.12 ± 2.71	0.32 ± 2.14	0.88 ± 1.81	1.12 ± 2.26
[Hz]	TP13-TP7	2.03 ± 3.10/*	2.77 ± 3.96/*	3.20 ± 2.78/***	4.85 ± 3.95/***
	TP13-TP3	3.15 ± 3.47/***	3.09 ± 4.45/***	4.08 ± 2.30/***	5.97 ± 5.05/***

*p < 0.05, **p < *α*_HRV_ ( = 0.01), ***p *<* 0.001.

**Table 3:** Change in SC, P_ET_CO_2_, and the fNIRS variables for the L-PCF and the R-PCF with respect to the colour conditions. Refer to Table 2 for a detailed description.

		Mean change ± SD of variable/Result of Friedman Test			
		red	green	blue	yellow
SC	TP7-TP3	0.05 ± 0.19/*	0.08 ± 0.85	0.07 ± 0.37	0.28 ± 0.82
[μS]	TP13-TP7	0.57 ± 0.94/**	0.41 ± 0.54	0.76 ± 0.94/***	1.48 ± 2.58/***
	TP13-TP3	0.62 ± 0.92/***	0.53 ± 0.95/*	0.83 ± 0.91/***	1.80 ± 3.00/***
P_ET_CO_2_	TP7-TP3	−0.29 ± 1.44	0.24 ± 1.09	−0.27 ± 0.98	−0.63 ± 1.45
[mmHg]	TP13-TP7	−1.11 ± 1.68/**	−0.88 ± 1.29	−1.14 ± 1.28/***	−1.23 ± 1.24/**
	TP13-TP3	−1.40 ± 1.90/***	−0.64 ± 1.50	−1.40 ± 1.51/***	−1.86 ± 1.90/***
L-PFC [O_2_Hb]	TP7-TP3	0.30 ± 0.98	0.28 ± 0.97	0.59 ± 1.22	0.90 ± 0.94/**
[μM]	TP13-TP7	0.79 ± 0.82/***	0.96 ± 1.84/*	0.87 ± 1.61/*	0.83 ± 1.20/**
	TP13-TP3	1.09 ± 1.19/***	1.24 ± 1.94	1.46 ± 2.63/**	1.73 ± 1.39/***
L-PFC [HHb]	TP7-TP3	−0.19 ± 0.54	−0.14 ± 0.70	−0.20 ± 0.51	−0.27 ± 0.27/**
[μM]	TP13-TP7	−0.25 ± 0.79/***	−0.14 ± 0.54/*	−0.06 ± 0.67	−0.10 ± 0.42
	TP13-TP3	−0.44 ± 0.73/***	−0.28 ± 0.59/**	−0.26 ± 0.99	−0.37 ± 0.49/**
L-PFC [tHb]	TP7-TP3	0.13 ± 0.98	0.13 ± 0.75	0.39 ± 0.98	0.66 ± 0.89
[μM]	TP13-TP7	0.53 ± 0.77/**	0.80 ± 1.67/*	0.79 ± 1.71	0.74 ± 1.02/**
	TP13-TP3	0.66 ± 1.21/**	0.94 ± 1.99	1.18 ± 2.50	1.40 ± 1.40/***
L-PFC StO_2_	TP7-TP3	0.39 ± 1.35/*	0.45 ± 1.24	0.51 ± 1.29	0.89 ± 0.91/**
[%]	TP13-TP7	0.93 ± 1.46/***	0.91 ± 1.25/**	1.11 ± 2.05	0.46 ± 1.32
	TP13-TP3	1.33 ± 1.33/***	1.37 ± 1.09/***	1.62 ± 2.47/**	1.35 ± 1.57/***
R-PFC [O_2_Hb]	TP7-TP3	0.16 ± 1.33	0.45 ± 0.58/*	0.19 ± 1.08	−0.05 ± 1.01
[μM]	TP13-TP7	−0.35 ± 1.26	−0.35 ± 1.37	−0.32 ± 1.58	−0.17 ± 1.49
	TP13-TP3	−0.19 ± 2.16	0.10 ± 1.48	−0.13 ± 1.39	−0.22 ± 1.20
R-PFC [HHb]	TP7-TP3	−0.12 ± 0.74	−0.25 ± 0.72	−0.05 ± 0.81	0.25 ± 0.63
[μM]	TP13-TP7	0.32 ± 0.65/*	0.54 ± 1.20/*	0.36 ± 1.11	0.58 ± 1.89
	TP13-TP3	0.19 ± 0.72	0.29 ± 1.35	0.31 ± 1.00	0.83 ± 2.07
R-PFC [tHb]	TP7-TP3	−0.02 ± 1.15	0.13 ± 0.53	0.16 ± 1.12	0.18 ± 1.05
[μM]	TP13-TP7	0.04 ± 1.35	0.22 ± 0.70	0.01 ± 0.86	0.40 ± 1.18
	TP13-TP3	0.01 ± 2.31	0.34 ± 0.75	0.16 ± 1.53	0.59 ± 1.75
R-PFC StO_2_	TP7-TP3	0.28 ± 1.66	0.73 ± 1.43	0.26 ± 1.40	−0.37 ± 1.55
[%]	TP13-TP7	−0.86 ± 1.61/*	−1.12 ± 2.71	−0.69 ± 2.59	−0.62 ± 3.04
	TP13-TP3	−0.58 ± 2.15	−0.40 ± 3.08	−0.43 ± 2.06	−0.98 ± 2.74

*p < 0.05, **p < *α*_NIRS_ (=0.01) or *p* < *α*_CO2_ (=0.05) or *p* < *α*_SC_ (=0.03), ***p < 0.001. **refer to significance after FDR correction, α_CO2_ applies to the P_ET_CO_2_ data, *α*_SC_ to the SC data, and *α*_NIRS_ to the NIRS data.

## Discussion

Light of different colours altered brain physiology. We observed that especially yellow light exposure evoked changes in [O_2_Hb] and [HHb] concentrations in the L-PFC, which correspond to the typical activation responses in fNIRS^49^. Although the time scale is longer (10 min), this could indicate a functional brain response to yellow light exposure. We additionally observed an increase in StO_2_ during red light exposure, but no changes due to blue light exposure, as reported by Weinzirl *et al*.^37^. Contrary to Weinzirl *et al*. we employed LED (light emitting diode) sources whereas Weinzirl *et al*. used incandescent light and colour filters (LEE Filters Hampshire, UK). In addition, the light intensities employed in the study of Weinzirl *et al*. were different for the involved colours red and blue (65 and 25 lux at eye level, personal communication). The different light sources have different light spectra which in turn may affect brain physiology differently, e.g. via the non-image forming system^50, 51^. Furthermore, less subjects contributed to the yellow light changes (12 instead of 16) and yellow light was a composition of green and red light, i.e. having two peaks in the spectrum. Although the illuminance was always 30 lx, the broader spectral response may have had better efficacy. This assumption derives from the involvement of the rods and cones in the non-image forming system^19, 52, 53^.

We observed the changes due to yellow light only in the L-PFC but not in the R-PFC, which may indicate that the response to coloured light is hemisphere-dependent. No similar investigations have been found in the fNIRS literature, but in EEG literature, it was reported that alpha power decreased after 5 min of blue or red light exposure and the decrease was stronger under red light in the parieto-occipital brain^33^. Relative alpha power increased in the frontal-central brain^54^ and the alpha attenuation coefficient decreased (parietal)^42^ with decreasing lamp colour temperature. Relative alpha power, but not beta power, was also increased in green light relative to red and blue light in parietal derivations^55^. Hence, the EEG studies have reported quite different effects when measuring from different brain regions. This supports our notion, that coloured light exposure may induce localized brain responses.

Changes of skin conductance due to coloured stimuli have been reported in the literature^20, 44, 45^. We observed an increase in tonic skin conductance for red, blue and yellow light, specifically occurring after the light exposure. Similarly, HR increased for all colours and P_ET_CO_2_ decreased during recovery (except for green). The systemic changes in the later recovery may have been caused by discomfort of the fNIRS sensors, which were placed on the subjects’ forehead. The observed changes in HRV variables and P_ET_CO_2_ reflect this pain experience, i.e. increase in SC, increase in HR, decrease in P_ET_CO_2_ (due to changes in respiration, weak pain-related hyperventilation). We are confident that the changes observed during the light exposure itself were not strongly affected by the pain-related confounding effect since the subjects reported that the discomfort started mainly in the recovery period. The left-right difference of the fNIRS responses also seems odd concerning the discomfort, since it remains unclear why it only would elicit responses in the L-PFC. Many studies did not measure systemic physiology and thus could not detect if the set-up or protocol per se induces changes, which might have led to misinterpretations of the results. We suggest that fNIRS measurements should always be complemented by systemic physiology measurements and that any discomfort of the subjects due to the measurement procedure must be avoided or at least mentioned.

In accordance to other studies^22, 43^, red light increased the LF/HF ratio (from approx. 3.2 to 4, median values) in our study, although the increase for the coloured light exposure per se was not significant after FDR correction. Additionally, an increase in LF/HF was observed due to green light in the recovery period. It may be just an interesting coincidence that we found LF/HF change in the recovery after green light exposure, where we hardly saw P_ET_CO_2_ and SC changes (but still HR changes), but no LF/HF changes in the recovery period otherwise. The above mentioned discomfort-activated sympathetic autonomous activity should also affect the HRV, especially the LF component^56^, and thus the LF/HF ratio. Although these results remain to be elucidated, the influence of red light on the LF/HF ratio and thus the autonomic nervous system may open new interesting fields of applications. Increase of LF/HF could be due to vagal activation and thus relaxation. The results from the MDMQ, which indicated higher sleepiness from red light, supports this notion.

On the other hand we did not see an increase in absolute HF power during blue light exposure, in comparison to a few studies that reported this effect^22, 25, 40, 41^. Other studies have not reported modulation of HF power due to blue light^23, 42^. Contrarily, absolute HF power decreased for the whole time series. Different light spectra or other differences, like controlled breathing^40^ or higher illuminance^22, 25, 40^ may be sources of the found discrepancies.

We did not find interactions between the fNIRS variables with the systemic modalities during the coloured light exposure. The fNIRS variables changed during yellow light exposure but variables of the systemic physiology hardly changed at all for the yellow condition.

We observed a high inter-subject variability of the physiological changes (see the large error bars in Figs. 1 and 2). This indicates that each subject showed different responses to the experimental paradigm and that a group-based analysis is only one aspect of analysing and interpreting the data. A complementary subject-specific analysis is indicated and will be reported in the future.

Since LED light is expected to become the standard room lightning source, in this study LED light was chosen and investigated. Nevertheless, it is worthwhile to also research other light sources such as fluorescence and metal halide lights as well as natural light sources such as the sunlight in the future.

### Conclusion

We studied the effect of coloured light on skin conductance, P_ET_CO_2_, HRV, and brain haemodynamics. Our results indicate a brain activation of the left pre-frontal brain especially due to yellow light and a LF/HF increase due to red light exposure, which is in line with the literature.

## Methods

### Subjects

Seventeen healthy, right-handed subjects were assessed (6 female; median age 29 years, range 25–65 years). One subject was excluded from the data analysis because of red-green colour blindness, according to the Ishihara colour blindness test^57^. The study was approved by the Ethics Committee of the Canton of Zurich and subjects signed informed consent prior to the participation. The study was conducted and the methods in it were applied according to the declaration of Helsinki^58^ and to the essentials of good epidemiological practice^59^.

### Protocol

The rationale for the protocol in investigating effects of coloured light was to utilize LED lights because they are expected to become the standard light sources for room lightning in the future including their capability to emit coloured light and because they are ideally suited for reproducible coloured light emission. Long periods of exposure were chosen also because they resemble the situations in daily life.

Each subject was exposed to light of four different colours (red, green, blue, yellow) for ten minutes on four different days. The colour order was randomized. Four subjects were not exposed to yellow light. Before (baseline, 8 min) and after (recovery, 15 min) the coloured light exposure, subjects were in darkness. We intentionally did not include multiple blocks of light exposure, because we wanted to create an everyday-like (artificial) lighting situation and because we could compare our results to previous literature in that way. They were always sitting upright in a comfortable reclining chair with the feet on the ground and the head was supported by a pillow. The subjects faced a white wall (distance eyes-wall ~110 cm). The illuminance was 30 lux at eye level. Measurements were carried out between 8:30 am and 6:00 pm. Additionally, before and after the experiment, the participants were asked to fill in the German paper version of the MDMQ (short form A)^60^.

### Coloured light exposure

Illumination of the white wall was achieved by four RGB LED PAR 56 light cans (Cameo CLP56RGB05, Adam Hall GmbH, Germany). The four light beams were directed onto a white wall in such that light was distributed evenly on the wall. Peak wavelengths for the three different lamp LEDs were: Red: ~630 nm (full width at half maximum FWHM ~20 nm), green: ~515 nm (FWHM ~32 nm), blue: ~450 nm (FWHM ~20 nm) Yellow was mixed from red (78.5%) and green (21.5%).

### Functional near-infrared spectroscopy

Functional brain activation was measured by a frequency-domain multi-distance NIRS system (ISS Imagent, ISS Inc., Illinois, USA). It sampled tissue absorption and scattering coefficients at two wavelengths (760 and 830 nm) at 25 Hz. Standard ISS sensors with a line geometry were employed, which measure at four distances (approx. 2, 2.5, 3, 3.5 cm). The optodes were placed on the left and right side of the forehead over the pre-frontal cortex at Fp1-F3/Fp2-F4 according to the international 10–20 system^61^.

The signals [O_2_Hb], [HHb], [tHb] and StO_2_ were calculated by the AC and phase algorithm as described by Fantini *et al*.^62^ and Hueber *et al*.^63^. For convenience we created an own script in Matlab, which delivers the same values as the proprietary ISS software.

Haemoglobin concentration changes were calculated by the proprietary standard ISS software using AC and phase^63^. Four haemoglobin concentration pairs, which consisted of oxygenated and deoxygenated haemoglobin ([O_2_Hb] and [HHb]), were obtained from the left and right pre-frontal (L-PFC, R-PFC) and occipital cortex. Only data from the pre-frontal regions are presented here. Total haemoglobin and tissue oxygen saturation were calculated as [tHb] = [O_2_Hb] +[HHb] and StO_2_ = [O_2_Hb]/[tHb], respectively.

### Skin conductance

A wired electro-dermal activity measurement system (Verim Mind-Reflection GSR, Poland; sampling rate: 2 Hz, 16 bit resolution, measurement range: 10 kΩ to 4.5 MΩ) was employed to measure skin conductance. The skin-conductance electrodes were placed on the proximal parts of the participant’s ring and middle fingers. The sampling rate was 2 Hz. Data were converted to μS and then processed with Ledalab, a Matlab toolbox which extracts the phasic and tonic skin-conductance components by continuous decomposition analysis (CDA)^64^. The toolbox and further details about it are available on the Ledalab website (www.ledalab.de). In short, the CDA enables to decompose the skin conductance signal in two components, one with fast changes (i.e. the phasic part) and the other with the overall long-term trend (i.e. the tonic part). CDA conducts the decomposition by performing four signal processing steps: first, the tonic component is estimated, then a non-negative deconvolution of the phasic skin conductance data is applied, followed by a segmentation of signal drivers and reminders, and finally the reconstruction of the skin conductance data^64^. For our analysis we used standard values of LedaLab, which then optimizes the parameters of the CDA automatically. In the present paper we were interested in the long-term trend of the skin conductance, i.e. the tonic component, which was then used for the signal processing and statistical analysis.

### Partial pressure of carbon dioxide

The P_ET_CO_2_ sensor tube was fixed below the nostril of the participants with adhesive tape. The Normocap CO_2_ monitor (Datex Instrumentarium Oy, Finland) was connected analogously to the ISS auxiliary input. The auxiliary input was a 5 V TTL signal and thus the absolute calibration was lost. We empirically determined conversion factors to convert the TTL signal into units of mmHg for the CO_2_ partial pressure. We calculated P_ET_CO_2_ from the peak maxima of the P_ET_CO_2_ time course. One data point was obtained for each breathing cycle. The resulting P_ET_CO_2_ time course was interpolated to 1 Hz by a piecewise cubic spline and then smoothed by a robust moving average (Matlab smooth function with ‘rloess’ option) with a window length of 1 min. The robust version of the smoothing procedure applies zero weight to outliers.

### Heart-rate variability

An electrocardiogram (ECG) was recorded by a standard Holter ECG system (Schiller Medilog AR4plus) at a sampling rate of 1 kHz and using four derivations. The R-R intervals (i.e. the temporal distance between two consecutive R-peaks of the ECG QRS-complex) were already detected by the systems software and then exported to Matlab. We interpolated the R-R intervals to 1 Hz by linear interpolation. According to the NIRS data processing we divided the time series in thirteen 150 seconds segments. For each of these segments we calculated SDNN, RMSSD, absolute VLF (0–0.04 Hz), LF (0.04–0.15 Hz) and HF (0.15–0.4 Hz) power, total power (0–0.4 Hz), normalized LF (LF/(LF + HF)) and HF (HF/(LF + HF)) power, and the LF/HF ratio, all according to ref. 56. We employed Welchs methods with two non-overlapping 75 s windows to compute the Fourier transform of the ECG equidistant sampled signals. Powers for each of the bands were calculated by summing up the respective frequency band and multiplying by the frequency resolution.

### Heart rate

Heart rate was calculated from the ECG data by simply calculating the inverse of the R-R intervals.

### Data analysis

Prior to analysis, too noisy data were rejected by manual inspection (StO_2_ outside 0–100%). This applied to three times series for the L-PFC and two time series for the R-PFC detector. Haemoglobin signals were low pass filtered (cut-off frequency: 0.3 Hz, optimized least squares finite infinite response filter with order 935) and then down-sampled to 1 Hz. Movement-related artefacts were removed employing the “movement-artefact removal algorithm” (MARA)^65^ with a slightly different reconstruction method. Instead of the spline interpolation we fitted a 2^nd^ degree polynomial model with a window length p to the artefact segments (by the Matlab function smooth with option ‘loess’), and then subtracted the fit from the artefact. Hence, *p* can be any positive integer value. The necessary parameters (*p*, length of moving window *L*, and the threshold *T*) were found individually for each haemoglobin time series. Most commonly, the length for the moving standard deviation window was *L* = 3 seconds and the artefact segments were not reconstructed but only modelled as a straight line by using *p* = 1 s. For 55.2% of the time series, no artefact correction was required, in 75%, the relative amount of movement artefacts was 0.3% or less, in 95%, it was 9.8% or less and the maximum amount of artefacts in a signal was 33.4%. To further denoise the data, they were low pass filtered again using a robust 2^nd^ degree polynomial moving average with a window length of 2 min. Finally, the signal was divided in thirteen 2.5 min segments and the median calculated for each segment. We choose the length of 2.5 min, because in the raw data we observed slow and continuous changes. Three segments resulted for the baseline (0.5–8 min, time points (TP) 1–3), four for the coloured light exposure (TP 4–7), and six for the recovery period (TP 8–13).

### Multidimensional mood-state questionnaire

From the MDMQ we calculated the three main scales elevated-depressed mood, wake-tired, and calm-nervous (minimum −8, maximum 8 points)^60^. To obtain a change in these scales, we calculated the differences of the scores before and after the measurement. The distribution of the data was not normal. The differences were tested by Wilcoxon signed rank tests for significance and by a Friedman test for a main effect of colour on the three scales. Significance level was always set to 0.05 (two sided).

### Statistics

To test for an overall change of each variable over time we employed Friedman tests (non-normal distribution of the data), including all thirteen time points (*p*-values denoted *p*_all_). To further test if the change is related to the coloured light exposure or to the recovery period we tested either all coloured light exposure time points against the last baseline time point (TP 3–7, denoted *p*_col_) or all recovery time points against the last coloured light exposure time point (TP 7–13, denoted *p*_recov_) by Friedman tests. We additionally calculated Wilcoxon signed rank tests for each time point compared to the last time point of baseline (TP3), displayed by asterisks in the figures (*α* = 0.05, two-sided). To check for a colour dependence of the results we calculated Friedman tests for the difference of the respective variable between the end of recovery and the end of coloured light exposure (TP13-TP7) and between the end of the coloured light exposure and the end of baseline (TP7-TP3) (*p*_betwc_). We corrected for multiple comparisons using the FDR correction^48^ with the FDR *α* = 0.05. The FDR correction was carried out separately for each set of Friedman tests of independent outcome variable(s). Thus, the multiple comparison situation was corrected by applying the FDR correction to the different time points, the two measured locations and the four colours of light exposure. No FDR correction was applied to the Wilcoxon singed rank tests. Corrected alpha for all tests related to fNIRS was *α*_NIRS_ = 0.011, for all tests related to HRV *α*_HRV_ = 0.01, for all test related to SC *α*_SC_ = 0.03, one for all tests related to P_ET_CO_2_ *α*_CO2_ = 0.05. The sample size *N* for the tests was normally 16 for red, green, and blue, and 12 for yellow. Due to missing data the sample size was smaller for some tests, but never below 12 for red, green, and blue, or 9 for yellow.

## References

[1] Maisels MJ, McDonagh AF (2008). Phototherapy for neonatal jaundice. N. Engl. J. Med..

[2] Gross F, Gysin F (1996). Phototherapy in psychiatry: clinical update and review of indications. Encephale.

[3] Radeljak S, Zarkovic-Palijan T, Kovacevic D, Kovac M (2008). Chromotherapy in the regulation of neurohormonal balance in human brain–complementary application in modern psychiatric treatment. Coll. Antropol..

[4] Even C, Schroder CM, Friedman S, Rouillon F (2008). Efficacy of light therapy in nonseasonal depression: a systematic review. J. Affect. Disord..

[5] Lieverse R (2008). Bright light in elderly subjects with nonseasonal major depressive disorder: a double blind randomised clinical trial using early morning bright blue light comparing dim red light treatment. Trials.

[6] Strong RE (2009). Narrow-band blue-light treatment of seasonal affective disorder in adults and the influence of additional nonseasonal symptoms. Depress. Anxiety.

[7] Wirz-Justice A (2011). A randomized, double-blind, placebo-controlled study of light therapy for antepartum depression. J. Clin. Psychiatry.

[8] Cajochen C (2007). Alerting effects of light. Sleep Med. Rev..

[9] Chellappa SL, Gordijn MC, Cajochen C (2011). Can light make us bright? Effects of light on cognition and sleep. Prog. Brain Res..

[10] Revell VL, Skene DJ (2010). Impact of age on human non-visual responses to light. Sleep Biol Rhythms.

[11] Rea MS, Figueiro MG, Bierman A, Bullough JD (2010). Circadian light. Journal of circadian rhythms.

[12] Lockley, S. W. In *Encyclopedia of Neuroscience* Vol. 3 (ed Larry, R. S.) Ch. Circadian rhythms: Influence of light in humans, 971–988 (Academic Press, 2009).

[13] Hanifin JP, Brainard GC (2007). Photoreception for circadian, neuroendocrine, and neurobehavioral regulation. J. Physiol. Anthropol..

[14] Yasukouchi A, Ishibashi K (2005). Non-visual effects of the color temperature of fluorescent lamps on physiological aspects in humans. Journal of Physiological Anthropology and Applied Human Science.

[15] Duffy JF, Czeisler CA (2009). Effect of Light on Human Circadian Physiology. Sleep Med. Clin..

[16] Cajochen C (2005). High sensitivity of human melatonin, alertness, thermoregulation, and heart rate to short wavelength light. J. Clin. Endocrinol. Metab..

[17] Munch M (2006). Wavelength-dependent effects of evening light exposure on sleep architecture and sleep EEG power density in men. Am. J. Physiol. Regul. Integr. Comp. Physiol..

[18] Morita T, Teramoto Y, Tokura H (1995). Inhibitory effect of light of different wavelengths on the fall of core temperature during the nighttime. Jpn. J. Physiol..

[19] Morita T, Tokura H (1996). Effects of lights of different color temperature on the nocturnal changes in core temperature and melatonin in humans. Appl. Human Sci..

[20] Jacobs KW, Hustmyer FE (1974). Effects of four psychological primary colors on GSR, heart rate and respiration rate. Percept. Mot. Skills.

[21] Abbas, N., Kumar, D. & McLachlan, N. The psychological and physiological effects of light and colour on space users. *Conf. Proc. IEEE Eng. Med. Biol. Soc*. **2**, 1228–1231, doi:10.1109/IEMBS.2005.1616646 (2005). 10.1109/IEMBS.2005.1616646 17282415

[22] Laufer L, Lang E, Izso L, Nemeth E (2009). Psychophysiological effects of coloured lighting on older adults. Lighting Res Technol.

[23] Schäfer A, Kratky KW (2006). The effect of colored illumination on heart rate variability. Forschende Komplementarmedizin.

[24] Xia Y-l, Shimomura Y, Katsuura T (2012). Comparison of the Effects of Wavelength and Intensity of Monochromatic Light on Cardiovascular Responses during Task and in Recovery Periods. Journal of the Human-Environment System.

[25] Edelhäuser F (2013). Impact of colored light on cardiorespiratory coordination. Evid. Based Complement. Alternat. Med..

[26] Morita T, Tokura H, Wakamura T, Park SJ, Teramoto Y (1997). Effects of the morning irradiation of light with different wavelengths on the behavior of core temperature and melatonin in humans. Appl. Human Sci..

[27] Katsuura T, Yasuda T, Shimomura Y, Iwanaga K (2007). Effects of monochromatic light on time sense for short intervals. J. Physiol. Anthropol..

[28] Okamoto Y, Nakagawa S (2015). Effects of daytime light exposure on cognitive brain activity as measured by the ERP P300. Physiol. Behav..

[29] An M, Huang J, Shimomura Y, Katsuura T (2009). Time-of-day-dependent effects of monochromatic light exposure on human cognitive function. J. Physiol. Anthropol..

[30] Okamoto Y, Rea MS, Figueiro MG (2014). Temporal dynamics of EEG activity during short- and long-wavelength light exposures in the early morning. *BMC Res*. Notes.

[31] Rahman SA (2014). Diurnal spectral sensitivity of the acute alerting effects of light. Sleep.

[32] Sahin L, Figueiro MG (2013). Alerting effects of short-wavelength (blue) and long-wavelength (red) lights in the afternoon. Physiol. Behav..

[33] Ali MR (1972). Pattern of EEG recovery under photic stimulation by light of different colors. Electroencephalogr. Clin. Neurophysiol..

[34] Vandewalle G (2007). Wavelength-dependent modulation of brain responses to a working memory task by daytime light exposure. Cereb. Cortex.

[35] Vandewalle G (2007). Brain responses to violet, blue, and green monochromatic light exposures in humans: prominent role of blue light and the brainstem. PloS one.

[36] Vandewalle G (2011). Effects of light on cognitive brain responses depend on circadian phase and sleep homeostasis. J. Biol. Rhythms.

[37] Weinzirl J, Wolf M, Nelle M, Heusser P, Wolf U (2012). Colored light and brain and muscle oxygenation. Adv. Exp. Med. Biol..

[38] Phipps-Nelson J, Redman JR, Schlangen LJ, Rajaratnam SM (2009). Blue light exposure reduces objective measures of sleepiness during prolonged nighttime performance testing. Chronobiol. Int..

[39] Lockley SW (2006). Short-wavelength sensitivity for the direct effects of light on alertness, vigilance, and the waking electroencephalogram in humans. Sleep.

[40] Mukae H, Sato M (1992). The effect of color temperature of lighting sources on the autonomic nervous functions. Ann. Physiol. Anthropol..

[41] Moharreri, S., Rezaei, S., Dabanloo, N. J. & Parvaneh, S. In *Computing in Cardiology*. (IEEE, 2014).

[42] Noguchi H, Sakaguchi T (1999). Effect of illuminance and color temperature on lowering of physiological activity. Appl. Human Sci..

[43] Litscher D, Wang L, Gaischek I, Litscher G (2013). The influence of new colored light stimulation methods on heart rate variability, temperature, and well-being: results of a pilot study in humans. Evid. Based Complement. Alternat. Med..

[44] Wilson GD (1966). Arousal Properties of Red Versus Green. Perceptual and motor skills.

[45] Nourse JC, Welch RB (1971). Emotional attributes of color: a comparison of violet and green. Percept. Mot. Skills.

[46] Su D, Liu C, Chiang C, Wang W (2012). Analysis of the long-term effect of office lighting environment on human responses. World Academy of Science, Engineering and Technology.

[47] Nunn JF, Hill DW (1960). Respiratory dead space and arterial to end-tidal carbon dioxide tension difference in anesthetized man. J. Appl. Physiol..

[48] Benjamini, Y. & Hochberg, Y. Controlling the false discovery rate: a practical and powerful approach to multiple testing. *Journal of the royal statistical society*. *Series B* (*Statistical Methodology*), 289–300 (1995).

[49] Villringer A, Planck J, Hock C, Schleinkofer L, Dirnagl U (1993). Near infrared spectroscopy (NIRS): a new tool to study hemodynamic changes during activation of brain function in human adults. Neurosci. Lett..

[50] Panda S (2003). Melanopsin is required for non-image-forming photic responses in blind mice. Science.

[51] Gaggioni G, Maquet P, Schmidt C, Dijk DJ, Vandewalle G (2014). Neuroimaging, cognition, light and circadian rhythms. Front Syst Neurosci.

[52] Hanifin JP (2006). High-intensity red light suppresses melatonin. Chronobiol. Int..

[53] Revell VL, Skene DJ (2007). Light-induced melatonin suppression in humans with polychromatic and monochromatic light. Chronobiol. Int..

[54] Iwakiri, K., Yasukouchi, A. & Murata, A. In *IEEE International Conference on Systems, Man, and Cybernetics*. 271–276 (IEEE, 1999).

[55] Yamashita, M., Yamada, I. & Yasuda, M. In *NES2012*. 5.

[56] Task Force of the European Society of Cardiology. Heart rate variability standards of measurement, physiological interpretation, and clinical use. *Eur. Heart J*. **17**, 354–381 (1996). 8737210

[57] Ishihara, S. *Tests for colour-blindness*. 24 plates edn, (Kanehara Shuppan Co., Ltd., 1972).

[58] World Medical Association. World Medical Association Declaration of Helsinki: ethical principles for medical research involving human subjects. *JAMA* **310**, 2191–2194, doi:10.1001/jama.2013.281053 (2013). 10.1001/jama.2013.281053 24141714

[59] Altpeter E (2005). Essentials of good epidemiological practice. Soz. Praventivmed..

[60] Steyer, R., Schwenkmezger, P., Notz, P. & Eid, M. *Der Mehrdimensionale Befindlichkeitsfragebogen. Handanweisung*. (Hogrefe-Verlag, 1997).

[61] Jurcak V, Tsuzuki D, Dan I (2007). 10/20, 10/10, and 10/5 systems revisited: their validity as relative head-surface-based positioning systems. Neuroimage.

[62] Fantini S, Franceschini MA, Gratton E (1994). Semi-infinite-geometry boundary problem for light migration in highly scattering media: a frequency-domain study in the diffusion approximation. Journal of the Optical Society of America B.

[63] Hueber, D. M. *et al*. New optical probe designs for absolute (self-calibrating) NIR tissue hemoglobin measurements. **3597**, 618–631, doi:10.1117/12.356784 (1999).

[64] Benedek M, Kaernbach C (2010). A continuous measure of phasic electrodermal activity. J. Neurosci. Methods.

[65] Scholkmann F, Spichtig S, Muehlemann T, Wolf M (2010). How to detect and reduce movement artifacts in near-infrared imaging using moving standard deviation and spline interpolation. Physiol. Meas..

